# Remembering the Leaders of China

**DOI:** 10.3389/fpsyg.2016.00373

**Published:** 2016-03-30

**Authors:** Mingchen Fu, Yan Xue, K. Andrew DeSoto, Ti-Fei Yuan

**Affiliations:** ^1^Faculty of Education, The University of Hong KongHong Kong, China; ^2^School of Psychology, Nanjing Normal UniversityNanjing, China; ^3^Association for Psychological ScienceWashington, DC, USA

**Keywords:** collective memory, recall memory, semantic memory, history, China

## Abstract

In two studies, we examined Chinese students’ memory for the names of the leaders of China. In Study 1, subjects were cued with the names of periods from China’s history. Subjects listed as many leaders as possible from each period and put them in the correct ordinal position when they could (see Roediger and DeSoto, 2014). Results showed that within each period, a primacy effect and sometimes a recency effect emerged. Moreover, the average recall probability for leaders within a specific period was a function of the ordinal position of the period. In Study 2, we asked another group of subjects to identify the sources through which they were able to recall each leader. We found that most subjects remembered leaders due to class and coursework. We also found a relation between a leader’s recall probability and the amount of information available on that leader on the Internet. Our findings further imply that the serial position function captures the form of collective memory.

## Introduction

The study of collective memory, defined as a form of memory that is shared by a large group of people and that represents the group’s identity (Hirst and Manier, 2008; Wertsch and Roediger, 2008; Roediger and Abel, 2015), was initiated by the French sociologist Halbwachs (1992) in the 1920s. Since then, sociologists, historians, psychologists, and others have conducted research on this important topic (e.g., Olick and Robbins, 1998; Stone and Hirst, 2014). Until recently, most have studied collective memory through the use of humanistic and qualitative research methods.

In recent years, however, psychologists have begun to apply quantitative and statistical techniques to probe collective memory (e.g., Overstreet and Healy, 2011; Kelley et al., 2013, 2015; Rubin, 2014; Zaromb et al., 2014; Roediger and DeSoto, in press). The studies reported in this burgeoning literature have begun to reveal knowledge about the psychological mechanisms underlying collective memory. One major finding of these studies is the existence of the serial position effect for ordered list items in collective memory.

The serial position effect is a commonly observed pattern describing an individual’s ability to recall a series of items learned earlier. Probability of recall follows a bow-shaped (“U”-shaped) function based on the position in which each item was presented. Items presented at the beginning of a list of to-be-learned materials as well as those presented at the end are usually recalled with significantly higher probabilities than those in the middle (these are called primacy effects and recency effects, respectively; e.g., Murdock, 1962). Recent quantitative studies on collective memory have shown some evidence indicating that the collective memory of a group of individuals for a series of items, including lyrics, famous books and movies, and political figures, also follow this function (Overstreet and Healy, 2011; Kelley et al., 2013, 2015; Roediger and DeSoto, 2014).

In a recent paper, Roediger and DeSoto (2014; see also Roediger and Crowder, 1976; Crowder, 1993) began to establish principles of forgetting from collective memory and proposed methods that can be used to estimate collective forgetting objectively. In Roediger and DeSoto’s research, subjects were asked to recall as many of the 44 U.S. presidents as possible and to place them in the correct ordinal position when they could (e.g., Abraham Lincoln in position 16). Americans’ recall of the presidents generally followed a classic serial position function. Probability of recall was very high for the first president (George Washington) and declined until the 7th or 8th president, showing a primacy effect; the low recall probability for subsequent presidents, then, increased starting at the 8th or 9th most recent president and reached near 100% recall for the current president (Barack Obama), showing a recency effect. Abraham Lincoln was a frequently recalled outlier, perhaps due to his important association with the American Civil War.

Roediger and DeSoto’s (2014) study demonstrated the potential for the psychological study of “the transmission and retention of cultural knowledge based both on exposure and the mechanisms that shape human memory” (Rubin, 2014, p. 1059). Nevertheless, to examine the generalizability of the findings in Roediger and DeSoto’s research, further efforts are needed. Particularly, it is unclear whether the patterns found in Americans could extend to those of other nationalities. For instance, the history of China is longer and far more complex than the history of the U.S. Since China was initially unified by the first Emperor of Qin in 221 BCE – almost 2,000 years before the U.S. was established – it has been led by a number of feudal dynasties, the Republic of China (ROC), and, since 1949, the People’s Republic of China (PRC). These different periods have featured different forms of government, whereas America has been a federal republic since it was founded. Also, in China’s long history there have been 128 national leaders, compared to America’s 44 presidents.

Thus, this research aimed to investigate the collective memory of Chinese people for the national leaders of China’s 2000-year history and extend Roediger and DeSoto’s (2014) research to a Chinese context. Given Americans’ collective memory for U.S. presidents, we supposed that Chinese subjects’ recall of national leaders from Chinese history would also show a serial position function. However, given the length of time, diversity of historical periods, and number of leaders that characterize China, it was expected that the collective memory of Chinese populations for their leaders would show a more complex pattern than the one shown for U.S. presidents. For instance, each of China’s distinct historical periods (e.g., Qin Dynasty) can also be considered specific units or chunks in collective memory.

Accordingly, we proposed two primary hypotheses:

(1) Within each specific historical period (e.g., Tang Dynasty, PRC), subjects’ recall of each national leader would follow the serial position function (i.e., earlier and later leaders within periods would be remembered better than intermediate leaders);
(2) The average recall probability for national leaders from each historical period, averaged within periods, would also follow a serial position function across periods (i.e., leaders from earlier and later periods, on average, would be remembered better than leaders from intermediate periods).

To investigate these hypotheses, we conducted two studies. Study 1 examined Chinese university students’ collective memory for the names of national leaders from Chinese history. In Study 2, we attempted to replicate and extend the results of Study 1. In addition, Study 2 also examined the sources through which Chinese students remembered these national leaders as well as the relationship between the community environment and collective memory.

## Study 1

### Method

#### Subjects

Four hundred thirty-three students (312 women and 121 men) from Nanjing Normal University, a public university in mainland China, were recruited by course instructors. Subjects’ ages ranged from 18 to 24 years (mean age = 20.9). The study was approved by the Ethics Committee at Nanjing Normal University. All subjects gave written informed consent.

#### Materials, Design, and Procedure

Subjects wrote down the names of the national leaders in Chinese history on a questionnaire. The questionnaire included a section collecting demographic information (sex and age) and 10 groups of blank spaces, respectively, corresponding to the 10 different periods in Chinese history. We provided the name of each historical period (e.g., Qin Dynasty) at the beginning of each group of blank spaces and numbered the lines corresponding to the number of leaders from the period. We also clarified in the questionnaire that the national leaders we referred to were the emperors of feudal dynasties and the presidents of the ROC and PRC.

We excluded the historical periods in which the nation was divided by a number of regimes and no national leader was identified (i.e., the Northern and Southern Dynasties Period, 420–589; and the Five Dynasties and Ten Kingdoms Period, 907–960). There was no overlap between any two historical periods. Subjects had the opportunity to recall a maximum of 128 leaders correctly (i.e., 128 blank spaces were provided). We note that there have been disputes concerning the division between some historical periods and the ordinal position of national leaders. We constructed the questionnaire using two commonly used historical textbooks for Chinese university students majoring in history (Wan, 2006; Chinese Academy of Social Sciences, 2013). We also consulted historical experts for their suggestions on the questionnaire. Therefore, the questionnaire was consistent with the mainstream view of history in China.

We told subjects to recall as many national leaders from each historical period as possible (measuring free recall) and to place them into the correct ordinal position when possible (measuring ordinal position recall). The criterion for correct free recall of a leader’s name was whether a subject had written it down in any blank space following the leader’s corresponding historical period. The criterion for a correct ordinal position recall of a leader’s name was whether a subject had not only written it down but also placed it in the correct position according to the chronological ordinal position of the leader in the corresponding historical period. For example, if the name Mao Zedong was written in *any* blank space in the PRC period section, it was counted as a successful free recall of Mao Zedong. However, successful ordinal position recall of Mao Zedong was awarded only if that leader’s name was written on the first blank space in the PRC period section.

All subjects completed the task in a classroom under a researcher’s supervision. A small pilot study suggested that 8 min was enough time for recall of as many leaders as possible. Therefore, in Study 1, we provided subjects with 10 min to complete the questionnaire. We used JASP and Microsoft Excel to analyze data and create the figures for both Study 1 and Study 2.

### Results and Discussion

**FIGURE 1:**
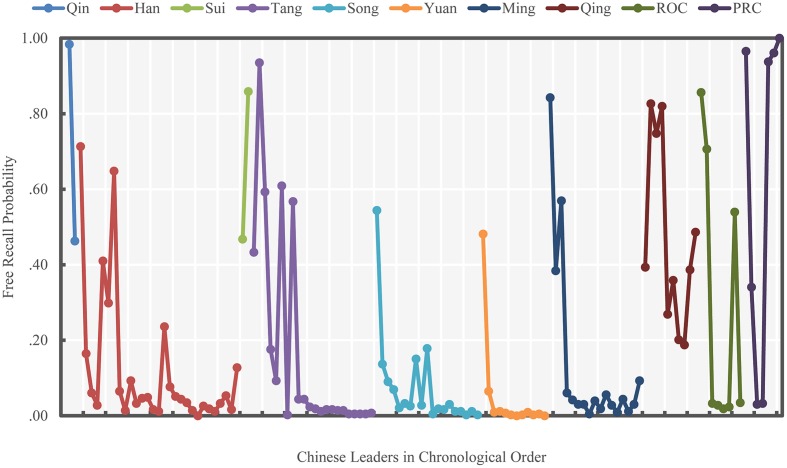
**Free recall probability of Chinese leaders (Study 1)**. Different colors indicate different periods of Chinese history.

**FIGURE 2:**
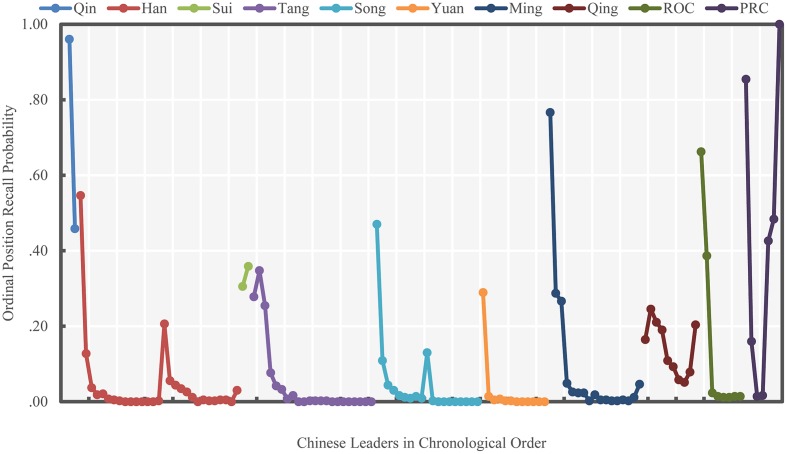
**Ordinal position recall probability of Chinese leaders (Study 1)**. Different colors indicate different periods of Chinese history.

The probabilities of successful free recall and ordinal position recall for each leader are shown in **Figures 1** and **2**, respectively. **Figure 1** reveals that the traditional bow-shaped serial position curve was found only for the Qing Dynasty and PRC, but not for other historical periods. However, we noticed that the free recall probability of the first leader of the Qing Dynasty was much lower than that of the second leader, which is not entirely consistent with a traditional primacy effect.

For the free recall probabilities in the rest of the historical periods (except Qin Dynasty and Sui Dynasty, which had only two national leaders in each), the primacy effect frequently occurred (i.e., the recall probabilities of first several leaders in each period were generally high); however, recency effects were absent.

In China’s different historical periods, some leaders recalled with notably high probabilities were not among the earliest national leaders in their corresponding periods (e.g., the 16th leader in Han Dynasty and the 7th leader of ROC were well remembered). Similar findings were also shown in Roediger and DeSoto’s (2014) study, which found that Abraham Lincoln was consistently recalled with a high probability even though he was neither an early nor recent U.S. president. Roediger and DeSoto suggested that the reason for this elevated recall was likely Lincoln’s distinctive influence on U.S. history. We suppose that, in our study, certain national leaders were more easily recalled because of the similar reasons. For example, the 7th leader of ROC – Chiang Kai-shek – had great influence in Chinese society because he was the leader of the Chinese government during the Anti-Japanese War (Second Sino-Japanese War) and the Chinese Civil War.

The ordinal position recall results, shown in **Figure 2**, present a much smoother and clearer pattern than does free recall. The overall pattern of correct ordinal position recall probabilities were largely consistent with the free recall results. Specifically, the primacy effect was observed at the beginning of each historical period, but only the recall probabilities within the Qing Dynasty and PRC resembled the serial position curve fully. Therefore, our first hypothesis was only partially supported, because the recency effect was absent in most historical periods.

**FIGURE 3:**
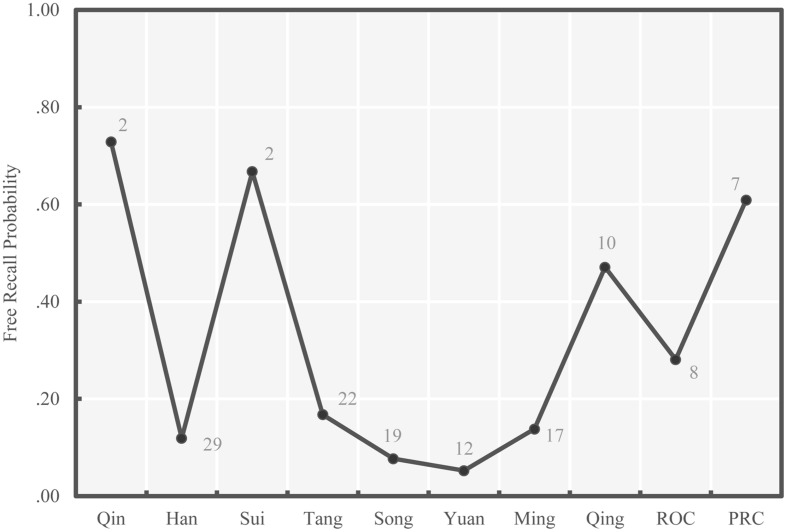
**Average free recall probability for each period of Chinese history (Study 1)**. The numbers of leaders in each period are presented beside each point.

**FIGURE 4:**
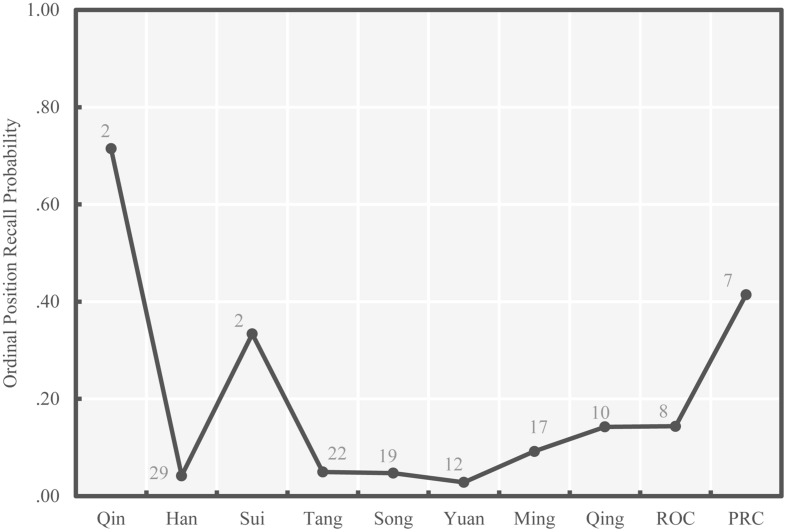
**Average ordinal position recall probability for each period of Chinese history (Study 1)**. The numbers of leaders in each period are presented beside each point.

The average probabilities of successful free recall and ordinal position recall of national leaders within specific historical periods, on average, are presented in chronological order in **Figures 3** and **4**, respectively. Consistent with our second hypothesis, patterns in both figures generally follow the bow-shaped curve. In both figures, the average recall probability for the second earliest historical period – Han Dynasty – was relatively low, much lower than the subsequent period – Sui Dynasty. This may be because the Han Dynasty had the largest number of national leaders (29) while Sui Dynasty had only two. A second deviation from the bow-shaped curve is found in **Figure 3**, which shows that leaders in the third recent historical period – Qing Dynasty – were recalled more readily than those in the second recent period – ROC. Hirst and Manier (2008) claimed that the collective memory of a group of people can be affected by the social environment. It is possible that Chinese university students’ collective memory for the ROC period might be to some extent reduced on account of political issues.

We note that there are challenges to interpreting **Figure 4**. Because each period featured different numbers of leaders, if a subject knew the period a leader belonged to, but not that leader’s ordinal position, the base rates of successful ordinal position placement by guessing would differ as a function of the leaders in the period. However, in this experiment we were not able to distinguish between subjects who were guessing both the period and position of leaders and subjects who knew the period but were guessing the position. Thus, we include the number of leaders in each period in **Figures 3** and **4** to assist with interpretation. The bow-shaped curve shown in **Figure 4** could be due to the lower numbers of leaders in the first and most recent periods.

In sum, Study 1 mostly supported our hypotheses, but also showed some unexpected findings.

## Study 2

We conducted a second study to replicate and extend the findings of Study 1. In Study 2, we asked subjects to recall the names of Chinese leaders, but we also queried subjects regarding the source of their memory for the leaders that were named. This information described how students were able to remember each leader. We also examined the social environment from which students may have learned that information by examining the number of Internet search hits for each of the different Chinese leaders, and relating that information to recall proportions.

### Method

#### Subjects

A second independent group of 247 students (185 women and 62 men) from Nanjing Normal University were recruited. Subjects’ ages ranged from 18 to 23 years (mean age = 20.8). The study was approved by the Ethics Committee at Nanjing Normal University.

#### Materials, Design, and Procedure

We explored the sources to which subjects attributed recall of national leaders via a questionnaire. In this study, we focused on memory for the 42 leaders from the four most recent historical periods – Ming Dynasty, Qing Dynasty, ROC, and PRC. Like in Study 1, we provided the names of each historical period and asked subjects to recall as many national leaders as possible and write their names down in the correct ordinal position when possible. Moreover, beside each name we asked subjects to choose the source from which they remembered the named leader. Four sources of recall were given as options: (1) history class, (2) non-fiction readings or lectures occurring out of class, (3) fiction in popular media (e.g., from novels or TV), or (4) “other sources.” Subjects were required to choose one to four sources. No subjects chose “other sources,” so this option was dropped from further analysis. All subjects answered the questionnaire in a classroom under a researcher’s supervision.

In addition, we also investigated the relationship between the quantity of information available on each leader on the Internet and memory for those leaders. We searched Baidu, the most popular internet search engine in China, and counted the number of search hits for each of the national leaders from the four periods of interest.

### Results and Discussion

Free recall probabilities of the 42 leaders in Study 2 were strongly correlated with those for the equivalent 42 leaders in Study 1, *r*(40) = 0.98, *p* < 0.001, replicating the results of Study 1.

**FIGURE 5:**
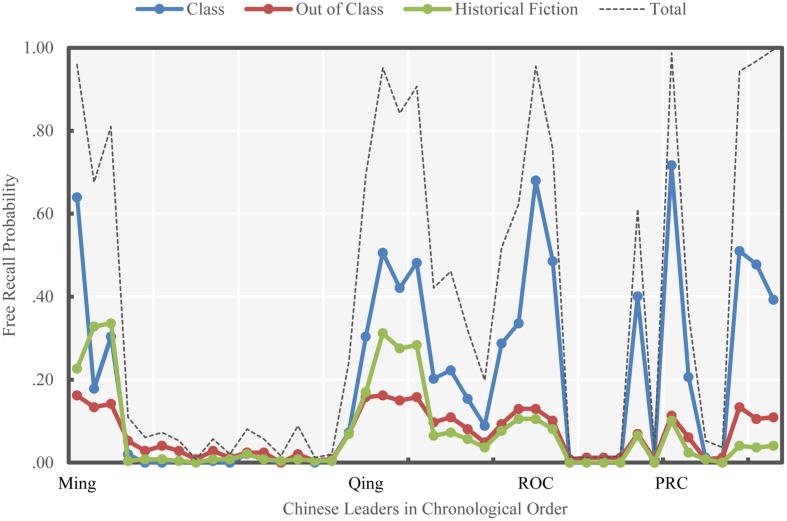
**Sources of free recall of Chinese leaders (Study 2)**.

**Figure 5** shows the rates with which subjects attributed their recall of each leader to each of the three sources. History class appeared to be the primary source measured through which subjects attributed their recall of national leaders, playing a much larger role than did the other two sources. A repeated-measures ANOVA confirmed this observation, finding statistically significant differences among the proportions of leaders reported to be retrieved via each source, *F*(2,82) = 18.80, *p* < 0.001. *Post hoc* paired-samples *t*-tests determined that recall of leaders, on average, was more likely to be attributed to learning from history class (*M* = 0.20) than out-of-class sources (*M* = 0.07), *t*(41) = 4.57, *p* < 0.001, or fiction (*M* = 0.07), *t*(41) = 4.36, *p* < 0.001.

Additionally, the correlations of the overall free recall probabilities with probability of recalling from class (Option 1), out of class (Option 2), and historical fiction (Option 3) were all strong (according to Cohen, 1992), with *r*(40) = 0.97, *p* < 0.001, *r*(40) = 0.93, *p* < 0.001, and *r*(40) = 0.71, *p* < 0.001, respectively, for each option. Study 2 showed similar unexpected findings as did Study 1: the distinctively high recall probability for Chiang Kai-shek, and the higher overall recall probabilities for national leaders in the Qing Dynasty as compared to ROC.

**FIGURE 6:**
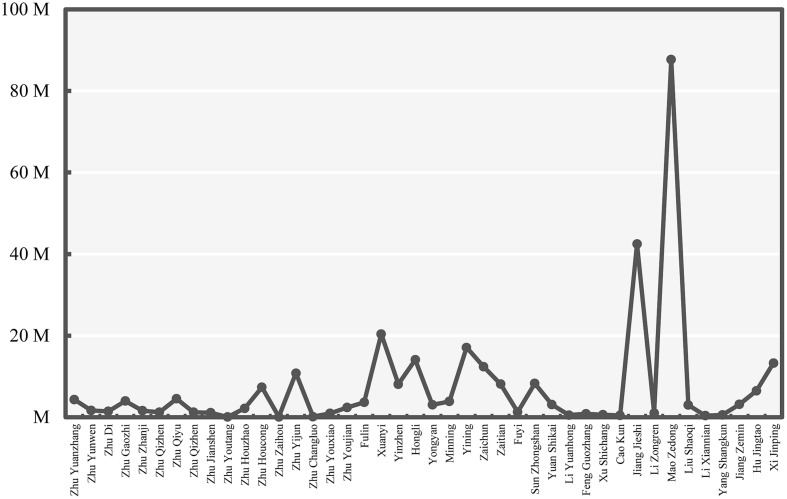
**Number of search hits on Baidu returned for each Chinese leader (from Ming Dynasty to PRC; Study 2)**.

With regard to the Internet environment, the number of search hits for each leader is shown in **Figure 6**. The number of hits for each leader was correlated with free recall probability of that leader to a moderate degree, *r*(40) = 0.41, *p* = 0.006. The pattern of the search hits of the national leaders appeared different from the pattern for free recall (as shown in **Figure 5**). Nevertheless, we found that the Baidu search results may account for the unexpected findings in Study 1. First, the number of search hits for Chiang Kai-shek (Jiang Jieshi) was significantly higher than the all other leaders except for the founder of the PRC government, Mao Zedong. Second, more information was available online for the national leaders of the Qing Dynasty (*M*_number of search hits_ = 9,205,000) than for those in ROC (*M*_number of search hits_ = 7,164,250) through Baidu.

In conclusion, the findings of Study 2 show that students attributed successful recall of Chinese leaders mostly to class and coursework, and less so to experiences occurring outside of the classroom, at least in regards to the four most recent periods of Chinese history.

Additionally, the Internet did appear linked to subjects’ collective memory for the national leaders of Chinese history to some degree. Rather than predicting the overall pattern of recall, however, perhaps the Internet is one way to help identify reasons for outliers that deviate from the general pattern predicted by the serial position curve. These results, taken together, show the interplay between collective memories held by individuals, and collective memories endorsed by culture.

## General Discussion

In these two studies, we examined Chinese university students’ collective memory for national leaders from Chinese history and investigated the source of these memories. Consistent with Roediger and DeSoto’s (2014) research, Study 1 showed that collective memory can be described by generalizable characteristics and studied objectively. In particular, within the long and complex history of China, we found that the average recall probabilities for national leaders within each specific historical period also followed the serial position function based on the historical period’s chronological order in Chinese history.

However, unlike the findings for collective memory of U.S. presidents in Roediger and DeSoto’s (2014) research, the serial position curve was only shown in the recall probability pattern in two specific historical periods – Qing Dynasty and PRC, respectively. In other historical periods, only primacy effects were identified. Given that Qing Dynasty and PRC are both relatively recent historical periods, it may be implied that the time at which a series of events occurred may affect collective memory patterns.

Furthermore, we found in Study 2 that resources available on the Internet to Chinese university students may help account for unexpected findings in collective remembering. These results hint at the role of the community environment in collective remembering. The finding is consistent with previous research showing that public silence leads to forgetting (Stone et al., 2012; Stone and Hirst, 2014). We believe that more efforts are needed to detect further how the environment can affect collective memory patterns. Furthermore, we also suggest that the Internet is a potentially useful resource for investigating the community environment’s relationship to collective memory in modern society.

Last, we found in Study 1 that the hypothesized patterns emerged more clearly in ordinal position recall than they did in free recall. Similar results were also shown in Roediger and DeSoto’s (2014) research, which presented a much smoother pattern for the ordinal position recall probabilities than for free recall probabilities. Similar findings have also been reported in a study assessing memory for fight song lyrics, which found that memory for order showed serial position effects whereas free recall did not (Overstreet and Healy, 2011). Therefore, this paper and the prior research imply that order information and individual item information may follow different patterns.

As a last point, the literature suggests that there are two different viewpoints used to explain how collective memories are stored – “in the world” vs. “in the individual” (Hirst and Manier, 2008). Researchers who believe that collective memory is located in the world claim that collective memory is to a great extent a reflection of the information resources in a community (Irwin-Zarecka, 1994). Conversely, those who believe that collective memory is located in the individual insist that collective memory is simply shared memories of individuals, largely depending on the mechanisms underlying individual memory (e.g., Passerini, 1987; Schafer, 1994). Our research provides an initial suggestion that both types of collective remembering may be occurring for Chinese leaders. The serial position function, investigated mostly for individual memories, also describes memory for Chinese leaders in Studies 1 and 2. At the same time, however, it may be the case that Chinese society and culture also guided remembering to some extent.

## Conclusion

We found a complex pattern of collective remembering for Chinese national leaders among Chinese university students. Our findings suggest that collective memory largely follows the rules that govern individual memory, and that collective memory can be studied in an objective way. The absence of recency effects in collective memory for most historical periods of Chinese history needs further explanation, however. We also found that community environment may be considered as a potential account of the memory patterns that objective rules fail to predict. We hope that future research can validate and extend these findings in other populations outside of China.

## Author Contributions

MF, YX, KD, and T-FY designed the study, all authors but KD performed the study, and all authors analyzed the results and wrote the paper together. All authors have read and approved the final version of the manuscript.

## Conflict of Interest Statement

The authors declare that the research was conducted in the absence of any commercial or financial relationships that could be construed as a potential conflict of interest.
